# Plumage polymorphism in the black sparrowhawk (*Accipiter melanoleucus*) is strongly associated with the expression level of *agouti signaling protein*

**DOI:** 10.1093/jhered/esae068

**Published:** 2024-11-18

**Authors:** Edmund Rodseth, Arjun Amar, Petra Sumasgutner, Robert A Ingle

**Affiliations:** Department of Molecular and Cell Biology, University of Cape Town, Rondebosch, Western Cape, South Africa; FitzPatrick Institute of African Ornithology, University of Cape Town, Rondebosch, Western Cape, South Africa; FitzPatrick Institute of African Ornithology, University of Cape Town, Rondebosch, Western Cape, South Africa; Department of Behavioural and Cognitive Biology, Konrad Lorenz Research Centre, University of Vienna, Vienna, Austria; Department of Molecular and Cell Biology, University of Cape Town, Rondebosch, Western Cape, South Africa

**Keywords:** *Accipiter*, agouti, eumelanin, MC1R, melanogenesis, plumage morph

## Abstract

Melanin-based plumage polymorphisms in birds are often associated with mutations in the melanogenesis genes, notably the *melanocortin-1 receptor* (*MC1R*), but may also arise through changes in the expression of these genes. Here we investigate the molecular basis of plumage polymorphism in both adult and juvenile black sparrowhawks (*Accipiter melanoleucus*), an African raptor that occurs in two adult color morphs, light and dark, and also exhibits variation in juvenile plumage coloration. Our results confirmed that plumage differences in adult morphs were a result of differential deposition of eumelanin in their ventral contour feathers. No polymorphisms in the coding regions of the *MC1R* or the *agouti signaling protein* (*ASIP*) genes associated with adult color morph were identified. However, lack of pigmentation in the developing breast feathers of light morph birds was strongly associated with elevated *ASIP* expression, and concomitant downregulation of the downstream melanogenesis genes *microphthalmia-associated transcription factor* (*MITF*), *tyrosinase* (*TYR*), and *tyrosinase-related protein 1* (*TYRP1*). Variation in the rufous colored plumage of juveniles was found to be due to covariation in eumelanin and pheomelanin levels in dorsal and ventral contour feathers. As in adult birds, an inverse relationship between melanin pigmentation and *ASIP* expression was observed. This covariation between eumelanin and pheomelanin levels is not consistent with the pigment type-switching model of melanogenesis, where increased *ASIP* expression results in a switch from eumelanin to pheomelanin production. This highlights the need for caution when extrapolating results from model systems to other animals and the value of conducting research in wild species.

## Introduction

Phenotypic polymorphism is the presence of two or more distinct, genetically determined phenotypes in a population independent of sex, age, or seasonality at a frequency too great to be maintained by spontaneous mutation (Huxley 1955; Roulin 2004). While a topic of considerable interest in evolutionary biology, the genetic basis of phenotypic polymorphisms in wild populations is often unknown, hindering a full understanding of their evolution, maintenance, and adaptive significance (Roulin 2004; Mundy 2005; Orteu and Jiggins 2020). A common type of phenotypic polymorphism in birds is plumage color polymorphism, which has been reported in 3.5% of all bird species (Galeotti et al. 2003). The frequency of color polymorphism varies widely between orders and is particularly high in raptors where around 30% of species exhibit some form of polymorphic plumage (Fowlie and Krüger 2003; Galeotti et al. 2003; Chakarov et al. 2011). Some species exhibit continuous variation in plumage coloration, including the barn owl (*Tyto alba*), Swainson’s hawk (*Buteo swainsoni*), and common buzzard (*Buteo buteo*), (Krüger and Lindström 2001; Roulin and Dijkstra 2003; Briggs et al. 2011; Kappers et al. 2017), while in others, individuals occur in two or more discrete plumage morphs, such as the booted eagle (*Hieraaetus pennatus*), Eleanora’s falcon (*Falco eleonorae*), ferruginous hawk (*Buteo regalis*), and black sparrowhawk (*Accipiter melanoleucus*) (Schmutz and Schmutz 1981; Galván et al. 2010; Gangoso et al. 2011; Amar et al. 2013; Bosch et al. 2020).

Avian plumage coloration is primarily determined by melanin and carotenoid pigments (McGraw 2006). Melanin is found in two major forms, eumelanin, which is dark brown/black in color, and pheomelanin, which is red/yellow. Integumentary structures containing melanin show a variety of colors depending on the amount and type of melanin present, ranging from bright yellow to dark brown or black (Riley 1997; McGraw et al. 2004; Lin and Fisher 2007; Galván and Solano 2016). With the exception of porphyrins, which contribute to the reddish color of juvenile black-shouldered kites (*Elanus caeruleus*) (Negro et al. 2009; Negro and Galván 2018), only melanin pigments have been implicated in feather coloration in the diurnal birds of prey, suggesting that differences in the amount and type of melanin in feather tissue likely underlie most examples of plumage polymorphism in this group.

**Fig. 1.**
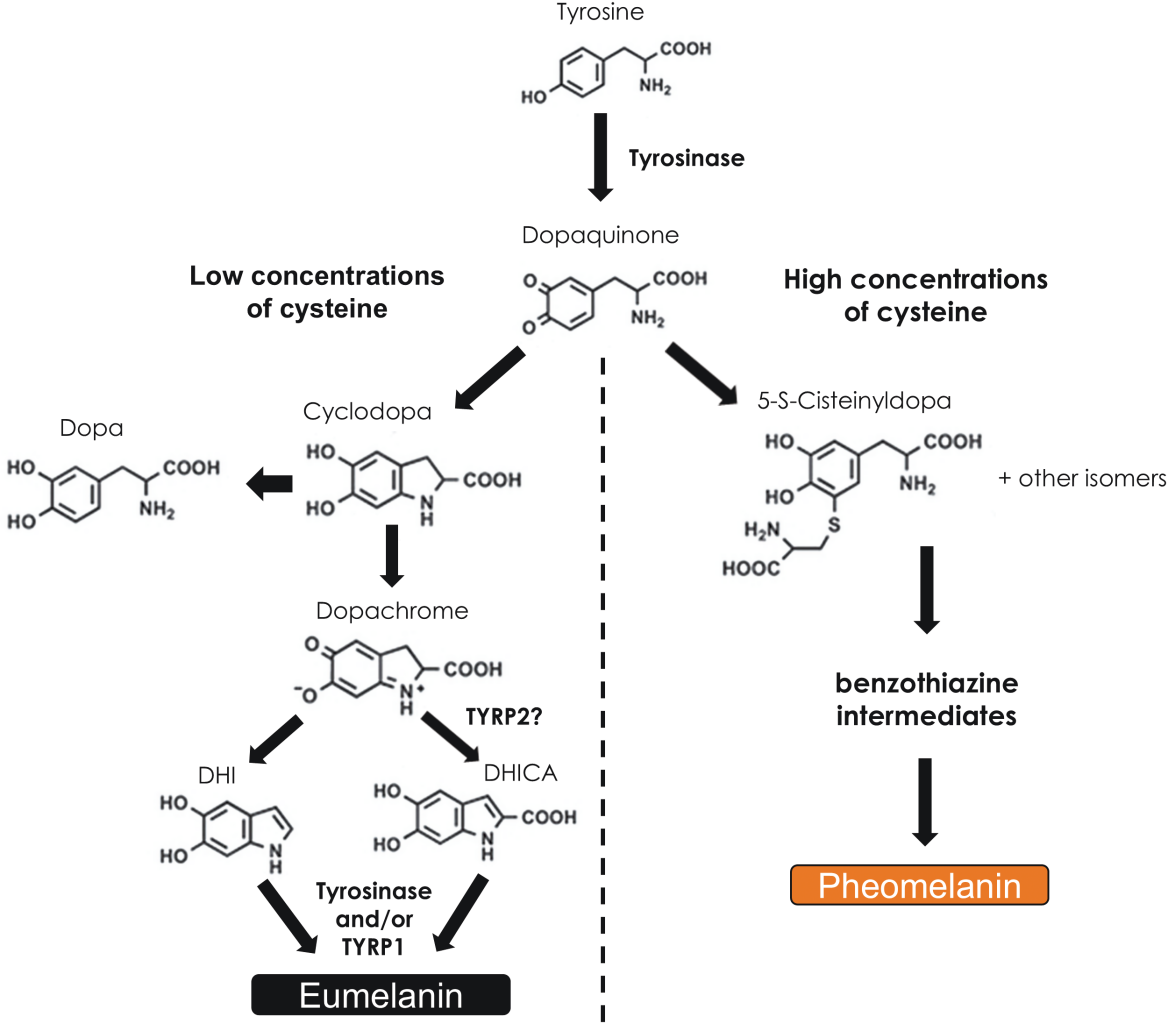
The major melanin biosynthesis pathway in birds, showing the eumelanin and pheomelanin production pathways. Tyrosine is oxidized to form dopaquinone by the enzyme tyrosinase. In the presence of high levels of Cys or other thiol-containing compounds, dopaquinone spontaneously reacts with them to form cisteinyldopas at a rapid reaction rate, which proceed spontaneously to form pheomelanins via benthothiazine intermediates. If levels of Cys are low, dopaquinone cyclizes at a slower rate to form dopa and cyclodopa. Cyclodopa then spontaneously reacts with dopaquinone to form dopachrome, which gives rise to 5,6-dihydroxyindole (DHI) and 5,6-dihydroxyindole-2-carboxylic acid (DHICA), possibly catalyzed by tyrosinase-related protein 2 (TYRP2). DHI and DHICA, along with their oxidized forms produced with the catalytic activity of tyrosinase and/or tyrosinase-related protein 1 (TYRP1), then polymerize to give rise to eumelanins.

Melanin is produced in specialized cells called melanocytes situated in the basal layer of the epidermis, with melanocytes in the feather follicles most relevant for determining plumage coloration in birds. Within melanocytes, melanogenesis (Fig. 1) takes place in melanosomes, lysosomal-like granules in which melanin is produced and packaged, which, when mature, are transferred to the cytoplasm of adjacent keratinocytes, where most pigment is ultimately found (Rees 2003; Lin and Fisher 2007). Both eumelanin and pheomelanin are derived from l-tyrosine, which is oxidized to dopaquinone by the enzyme tyrosinase (TYR) (Olivares and Solano 2009). The form of melanin that is subsequently synthesized depends primarily on the availability of the thiol-containing amino acid l-Cysteine (Cys) but is also influenced by *TYR* expression level and melanosome pH (Land and Riley 2000; Ancans et al. 2001; Lin and Fisher 2007; Ito and Wakamatsu 2008). When Cys levels are low, production of eumelanin is favored as dopaquinone will undergo spontaneous intramolecular cyclization to form cyclodopa. The final stages of eumelanin production require at least two enzymes, TYR and tyrosinase-related protein 1 (TYRP1) (Ito and Wakamatsu 2008; Galván and Solano 2016). Conversely, when Cys levels are high the synthesis of 5-*S*-cysteinyldopa from dopaquinone is favored (Jara et al. 1988; Ito and Wakamatsu 2008), and pheomelanin is then produced spontaneously without the catalytic activity of any other enzymes (Galván and Solano 2016).

Melanogenesis is under strong genetic control, and melanin-based pigmentation traits are highly heritable (Roulin and Ducrest 2013). The melanocortin-1 receptor (MC1R) pathway is the best-characterized melanogenesis regulatory pathway and has been frequently implicated in plumage variation in birds (Mundy 2005; Roulin and Ducrest 2013). The MC1R is a G-protein-coupled receptor that regulates the amount and type of melanin produced by the melanocyte. Activation of MC1R by the binding of α-melanocyte-stimulating hormone (α-MSH), leads to expression of the *microphthalmia transcription factor* gene (*MITF*), which in turn drives transcription of *TYR* and *TYRP1* required for eumelanin production (Cone et al. 1996). Thus, the activation of MC1R by α-MSH is believed to divert the melanocyte from the default production of pheomelanin to the production of eumelanin.

The MC1R also has an endogenous antagonist, agouti signaling protein (ASIP), which competitively inhibits αMSH binding (Lu et al. 1994). *ASIP* has been shown to be expressed in developing feather tissue in certain bird species and has been implicated in the switch from eumelanin to pheomelanin production in avian melanocytes (Hiragaki et al. 2008; Nadeau et al. 2008). However, *ASIP* expression in developing feathers can also lead to a lack of pigmentation, and regulation of the timing of *ASIP* expression in developing feather follicles may allow the formation of complex patterns in feathers (Lin et al. 2013).

Many examples of plumage polymorphism are associated with sequence variation in the *MC1R* gene. For example, a single non-synonymous difference in *MC1R* which leads to its constitutive activation is perfectly associated with a melanistic phenotype in the bananquit (*Coereba flaveola*) (Theron et al. 2001). The effects of variation in the *MC1R* can be diverse, as different alleles can be associated with differences in the amount of melanin pigmentation in each feather, as in the Arctic skua (*Stercorarius parasiticus*), or can cause differences in the distribution of dark plumage across the body, as in the snow goose (*Anser caerulescens caerulescens*) (Mundy et al. 2004). However, there are many cases where color polymorphism is not associated with *MC1R* sequence variation (see, for example, Cheviron et al. 2006; Dobson et al. 2012; Riyahi et al. 2015; Bam et al. 2019). Polymorphisms in other MC1R signaling pathway genes have also been linked to differences in plumage coloration in several species. For example, melanic birds from one island population of the chestnut-bellied monarch flycatcher (*Monarcha castaneiventris*) share a non-synonymous mutation in *ASIP* (Uy et al. 2016), while a premature stop codon in *MITF* results in white plumage in Japanese quail (*Coturnix japonica*) (Minvielle et al. 2010). Several recent whole-genome analyses have also demonstrated an association between variation at the *ASIP* locus and patterns of melanic plumage coloration among closely related populations or taxa, including warblers of the genus *Setophaga* (Baiz et al. 2021), the white wagtail (*Motacilla alba*) (Semenov et al. 2021), and capuchino seedeaters (genus *Sporophila*) (Estalles et al. 2022).

Plumage polymorphism can also be associated with localized patterns of differential expression of melanogenesis genes rather than polymorphisms in their coding regions. For example, *TYR*, *TYRP1,* and *MC1R* are expressed at lower levels in the gray torso of the hooded crow (*Corvus (corone) cornix*), compared to the carrion crow (*C. (corone) corone*), which has a black torso (Poelstra et al. 2014). Homozygosity for the *white MC1R* allele associated with lighter plumage coloration in barn owls correlates with increased expression of *ASIP* and reduced expression of melanogenesis genes, including *TYR* and *TYRP1*, as well as lower expression of *MC1R* itself (San-Jose et al. 2017). A negative correlation between *ASIP* mRNA levels and those of *TYR*, *TRYP1*, and *MC1R* has also been observed in tawny owl nestlings (Emaresi et al. 2013). Increased *ASIP* expression in Japanese quail was associated with a lighter, more pheomelanic phenotype (Nadeau et al. 2008), and *ASIP* expression has also been shown to correlate positively with pheomelanin levels in nestling barn swallows (*Hirundo rustica gutturalis*) (Arai et al. 2017).

Another common phenomenon involving differences in plumage coloration in birds is distinct juvenile plumage coloration. In the diurnal birds of prey, a particular pattern of delayed plumage maturation is common, with rufous plumage coloration in juveniles (likely rich in pheomelanin) which is absent or greatly reduced in the definitive adult plumage (Rodríguez-Martínez and Galván 2020). The juvenile plumage may also vary from pale to dark within a species as observed in the Ovambo sparrowhawk (*Accipiter ovampensis*), and black sparrowhawk (Ferguson-Lees et al. 2001; Rodríguez-Martínez and Galván 2020). Delayed plumage maturation in raptors may be explained by selective pressure on the plumage coloration itself, for example, if it acted as an honest signal of immaturity to conspecifics (Lyon and Montgomerie 1986; Hawkins et al. 2012), granted a greater degree of crypsis (Selander 1965; Rohwer et al. 1980), and/or promoted longer periods of parental care (Lawton 1986). It is also possible that pheomelanic plumage in juvenile birds results from the pleiotropic effects of the melanocortin system. The binding of melanocortin hormones and/or ASIP to four other melanocortin receptors (MC2-5R) in vertebrates is thought to explain why melanin-based coloration co-varies with a range of physiological and behavioral functions, including stress response, energy homeostasis, immune response, sexual activity, and aggressiveness (Ducrest et al. 2008). Distinct juvenile plumage coloration, or variation in juvenile plumage, might thus reflect a pleiotropic effect of altered melanocortin, or ASIP levels.

Alternatively, it has been proposed that pheomelanic juvenile plumage, especially among raptors, may have evolved to detoxify excess Cys (Galván et al. 2012), a semi-essential amino acid required for protein synthesis and production of the anti-oxidant glutathione (GSH). Excess Cys levels are associated with several physiological problems in birds, such as metabolic acidosis, thinning of eggshells, and poor growth (Galván et al. 2012; Galván 2017). As pheomelanin synthesis requires Cys, the deposition of pheomelanin into feathers could serve to reduce cellular Cys levels, thereby preventing toxicity (Galván et al. 2012; Galván 2017; Rodríguez-Martínez and Galván 2020). Supporting this hypothesis, pheomelanin production increased in response to supplementation of the diet of the Eurasian nuthatch (*Sitta europaea*) with Cys (Rodríguez-Martínez et al. 2019). It has been suggested that juvenile birds may be more prone to excess Cys accumulation since they remain in the less stressful environment of the nest (Vaanholt et al. 2008; Galván 2017). This effect could be particularly pronounced in carnivorous species due to the high protein content of a carnivorous diet (Rodríguez-Martínez and Galván 2020). Pheomelanic juvenile plumage has evolved independently numerous times in diurnal birds of prey and is more common in strictly carnivorous bird species than less strict carnivores or omnivores, independent of phylogeny (Rodríguez-Martínez and Galván 2020).

**Fig. 2.**
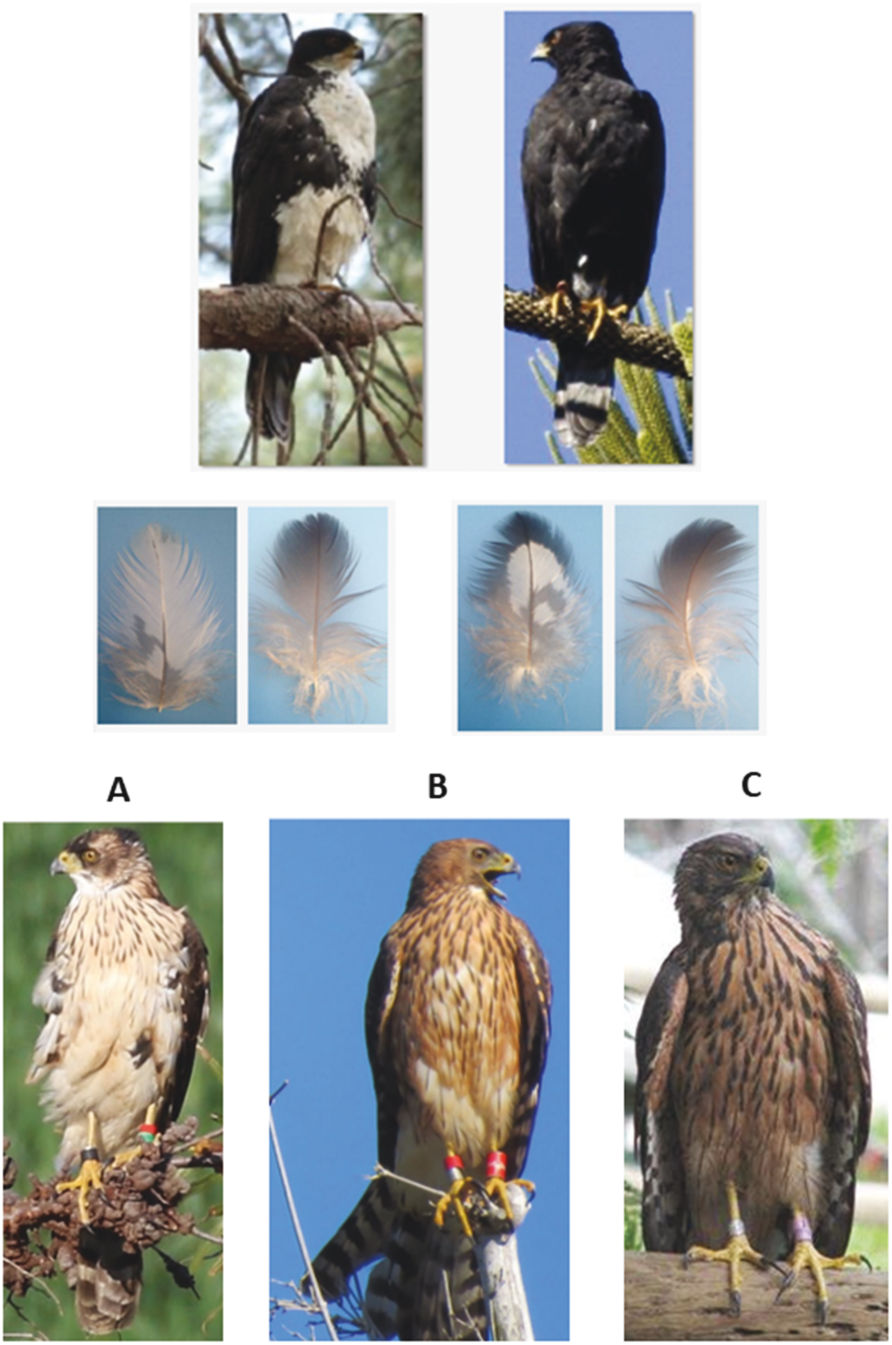
Plumage polymorphism in adult black sparrowhawks (top) and plumage variation in juvenile (under 1 year old) black sparrowhawks (bottom). Also shown are typical breast (left) and back (right) contour feathers from each adult morph. In juveniles, plumage varies from very pale breast feathers with faint dark streaking (A), through rufous breast feathers with more pronounced streaking (B) to dark rufous breast feathers with heavy streaking (C). Photo credits: A) Johann Strauss; B) Debbie Bentley-Buckle; C) Anne Koeslag.

The black sparrowhawk is a widespread sub-Saharan raptor and is an attractive model species in which to examine the genetic basis of both adult and juvenile plumage polymorphism. Adults occur in two morphs, light and dark, which differ in the extent of white plumage on the throat, chin, breast, and flanks (Fig. 2) (Amar et al. 2013). These two morphs are discrete, age invariant, and are inherited in a typical Mendelian manner, consistent with control by a single autosomal locus bi-allelic system, with dark dominant over light (Nebel et al. 2021b). Juvenile birds exhibit rufous plumage, which varies from pale to dark rufous (Fig. 2) and does not appear to be linked to adult plumage morph (Amar et al. 2013). This study aimed to determine whether plumage morphs in adult black sparrowhawks are associated with polymorphisms in the coding regions and/or differences in the expression of genes in the MC1R signaling pathway. We also analyzed the contributions of eumelanin and pheomelanin to juvenile plumage coloration in this species and tested whether variation in this phenotype was associated with differences in the expression of MC1R signaling pathway genes, parental morph, and/or environmental factors.

## Methods

### Ethics statement

Ethical clearance was obtained from the Science Animal Ethics Committee of the Faculty of Science, University of Cape Town (animal ethics clearance numbers A2/2014/2012/V37/AA and 2016/v11/AA). All necessary permits for working with wild birds and in national parks were obtained from Cape Nature and South African National Parks. Permission for work conducted on private property was obtained from the landowner. Captive adults were sampled with permission from their owners.

### Sampling of the study population

Birds were sampled between 2009 and 2019 across South Africa between 25.52°S to 34.70°S and 19.70°E to 33.00°E. Wild adults were trapped using bal-chatri traps (Berger and Mueller 1959) baited with live feral pigeons while nestling chicks were sampled directly from the nest. When more than one nestling was present, a single bird was chosen randomly for sampling. Nestlings were weighed, and tarsus length was measured using dial calipers. To assess the condition while accounting for differences in body size, the condition index was derived by extracting the residuals of a linear regression between body mass and tarsus length (Nebel et al. 2021a; Rodseth et al. 2024). This provides an effective method to quantify conditions while accounting for differences in body size (Reist 1985; Krebs and Singleton 1993; Jakob et al. 1996). Individuals were also examined for fault bars in their flight feathers, which could provide a proxy for stress during feather development (Jovani and Rohwer 2017).

One developing and two mature contour feathers were collected from the breast and mantle (upper back) feather tracts from 10 light morph and 10 dark morph adult birds. Two developing feathers were also collected from the same regions from 10 nestlings aged between 3.5 and 4.5 weeks old, the period during which they begin developing their juvenile plumage, making the collected feathers representative of their eventual juvenile plumage. Two developing breast feathers were collected from an additional 10 nestlings. Developing feathers were stored in RNAlater (Ambion) while in the field and transferred to −80 °C within 1 week of collection.

### Identification and cloning of coding regions of MC1R signaling pathway genes

Total RNA was isolated from developing feathers from all adults sampled and from 10 nestlings using acid guanidinium thiocyanate-phenol-chloroform extraction (Chomczynski and Sacchi 1987), and cDNA was synthesized using SuperScript III Reverse Transcriptase (Invitrogen). Avian homologs of *ASIP*, *MC1R*, *MITF*, *TYR, TYRP1,* and the reference gene *GAPDH* were identified in NCBI Genbank, aligned with BioEdit software (Hall 1998), and used to design primers to amplify full or partial coding sequences from black sparrowhawk cDNA (Supplementary Table S1). Target regions were amplified using the Kapa HiFi HotStart ReadyMix PCR kit (Kapa Biosystems) and the resulting polymerase chain reaction (PCR) products were cloned into pGEM-T-Easy (Promega) and sequenced on an ABI3730xl DNA analyzer (Applied Biosystems). The DNA sequences obtained were deposited in Genbank: *MC1R (*ON209113), *ASIP* (ON209114), *MITF* (ON209115), *TYR* (ON209116), *TYRP1* (ON209117), and *GAPDH* (ON209118).

### RT-qPCR analysis of gene expression

Primers for use in reverse transcriptase quantitative PCR (RT-qPCR) assays (Supplementary Table S2) were designed based on the DNA sequences obtained above. *GAPDH* was used as a reference gene as it displays stable expression and is a commonly used reference gene for feathers (Nadeau et al. 2008; Rodríguez-Martínez et al. 2019). The primers designed for *MITF* were specific to the *MITF-M* isoform which contains a melanogenesis-specific exon not found in other *MITF* isoforms. Primers were designed to span predicted intron-exon boundaries where possible. RT-qPCR was performed using the KAPA SYBR FAST qPCR Kit (Kapa Biosystems) in a Corbett Rotor-Gene 6000 HRM Real-Time PCR machine (Qiagen) with the following parameters: 1 × (95 °C for 3 min), 40 × (95 °C for 3 s, 60 °C for 20 s, 72 °C for 1 s), and 1 × (72 °C for 90 s). Only runs showing a single peak in the melt curve and where the efficiency of the standard curve was between 0.9 and 1.1 with an *R*^2^ value >0.99 were considered successful. Two technical replicates per biological sample were included and any samples where the technical replicates displayed a difference in *C*_*t*_ value >0.5 were re-run. The relative expression level of each gene was calculated using the standard curve method with normalization to that of the reference gene *GAPDH*.

### HPLC analysis of melanin content

High-performance liquid chromatography (HPLC) analysis was conducted to measure feather melanin concentration as described by Panzella et al. (2007), with minor modifications. Feather samples were trimmed to remove the barbules from the central rachis, washed twice in acetone, and dried. Samples of 5 mg of finely sliced adult feather tissue or 10 mg of juvenile feather tissue were added to 1 mL 1 M NaOH and treated with a final concentration of 1.5% (v/v) H_2_O_2_ at room temperature with vigorous shaking for 24 h. The samples were then treated with 200 μL 5% (w/v) Na_2_S_2_O_5_, adjusted to pH 4.0 with 85% (v/v) H_3_PO_4_, and filtered through 0.45 μm nylon syringe membranes prior to HPLC analysis. To generate a PTCA standard, 0.2 mg cuttlefish (*Sepia officinalis*) ink was subjected to the same procedure, since sepiomelanin (the melanin found in cuttlefish ink), consists of >98% eumelanin, and is widely used as a standard source of eumelanin. Furthermore, sepiomelanin is known to yield large amounts of PTCA by hydrogen peroxide degradation in an alkaline environment in the same manner as eumelanin found in pigmented tissues such as feathers (Palumbo 2003; Magarelli et al. 2010).

HPLC analysis was performed on an Agilent 1200 series instrument (Agilent) equipped with a 1200 thermostatted autosampler, 1200 Column thermostat, 1200 Quaternary pump, and a 1200 diode array detector set to 254 and 280 nm. A Synergi Hydro-RP 80A column (250 mm × 4.60 mm, 4 μm; Phenomenex) was used, with 1% (v/v) formic acid adjusted to pH 2.8 with NaOH/methanol 97:3 (v/v) as the eluant at a flow rate of 0.7 mL/min for 45 min per run. The injection volume was 50 μL per feather sample or 10 μL for the cuttlefish ink degradation mixture. Methanol/water 60:40 (v/v) was used at a flow rate of 0.7 mL/min for 15 min to wash the column between samples. Peaks were called, and their height and area were integrated automatically using the ChemStation for LC 3D systems (Rev 8.04.02) software (Agilent). Peaks corresponding to PTCA (degradation product of eumelanin) and BTCA (degradation product of pheomelanin) were identified by peak area ratios at 254 and 280 nm (A280/A254: PTCA = 1.1, BTCA = 0.6), elution times, and, for PTCA, comparison to the single, well-defined peak in the cuttlefish ink sample.

### Statistical analyses

All models were implemented in R version 3.5.3 (R Core Team 2019) with the MASS (Venables and Ripley 2002), lme4 (Bates et al. 2015), emmeans (Lenth 2023), effects (Fox 2003; Fox and Weisberg 2019), Hmisc (Harrell 2024), and car (Fox and Weisberg 2019) packages. All linear models with multiple predictor variables were followed by an analysis of variance (ANOVA) test implemented using the car package (Fox and Weisberg 2019). Where differences among groups were significant, we used post-hoc pairwise comparisons implemented using the emmeans package (Lenth 2023) to compare group means.

In adults, to explore whether eumelanin levels differed in breast and back feathers of light and dark adult morphs, we used a linear model with feather type (breast or back) within and between morphs (three levels: back for dark morph or light morph and breast for dark morph; light morph breast was excluded as no melanin was detected in these samples) as explanatory variables and relative PTCA levels as the response variable.

To explore the relative contribution of eumelanin and pheomelanin to juvenile breast feather coloration, a linear model was implemented with breast feather PTCA concentration (eumelanin) as the response variable and breast feather BTCA content (pheomelanin) as the explanatory variable.

In adults and juveniles, we explored whether the expression of the melanogenesis candidate genes differed by feather type (breast versus back—adults and juveniles) within and between morphs (light versus dark—adults only) using linear models with the following explanatory variables: feather type (juveniles) and feather type within and between morphs (adults, four levels: light morph breast and back, dark morph breast and back). For both models, the response variable was the relative expression of *ASIP*, *MC1R*, *MITF-M*, *TYR* or *TYRP1*. We initially explored the need to control for bird ID in the analyses given the potential lack of independence between observations from different feather types (breast and back) from the same bird by using linear mixed-effects models with morph and feather type as explanatory variables and relative expression of the melanogenesis genes as response variables with bird ID as a fixed effect, but we found no indication of an effect of ID, and so excluded this variable from the final analysis.

In juveniles, the expression levels of the five melanogenesis candidate genes in developing breast feather tissue were compared using correlation analysis. Pearson correlation coefficients between the expression levels of each pair of genes were calculated using the cor() function in R.

To explore the relationship between *ASIP* expression and BTCA and PTCA feather content in juvenile breast feathers, two linear models were implemented, with relative BTCA and PTCA content as response variables and relative *ASIP* expression as the explanatory variable. We explored whether parental morph is associated with juvenile plumage pigmentation by fitting either relative feather PTCA or BTCA levels in the juvenile as a response variable in a linear with the individual’s parental morph (dark–dark [DD], light–dark [LD], or light–light [LL]) as the explanatory variable. We also explored whether sex was associated with juvenile plumage pigmentation. Two linear models were implemented, with feather PTCA and BTCA content as the response variables and sex as the explanatory variable.

Finally, to explore whether body condition (condition index, CI) or number of fault bars observed in the flight feathers was associated with pheomelanin pigmentation two linear models were implemented, with feather BTCA content as the response variable and with either the number of fault bars or CI as explanatory variables.

## Results

### Adult plumage polymorphism is eumelanin-based and associated with differential expression of *ASIP* in developing feathers

Visual examination showed that dark morph breast contour feathers differ from feathers from light morph individuals by a variably sized dark patch on the tip and shaft of the feather, while the dorsal contour feathers of both morphs are wholly black (Supplementary Fig. S1). To confirm that the pigmentation of adult black sparrowhawks is indeed melanin-based, the melanin content of breast and back feathers from three dark and three light morph birds was quantified by HPLC (Table 1). No PTCA peak was identified in light morph breast feather samples, while the PTCA peak area in the breast and back feathers of dark morphs and the dark back feathers of light morphs were all found to be similar (*F*_2,6_ = 0.47, *P* = 0.66, adjusted *R*^2^ = −0.15). In contrast, no peak corresponding to BTCA was detected in any sample, indicating that adult feathers did not contain any pheomelanin, and thus coloration is entirely eumelanin-based.

**Table 1.** Mean PTCA peak area (mAU*s) ± standard deviation (*n* = 3) detected at 280 nm by HPLC analysis of alkaline hydrogen peroxide degradation mixtures containing 5 mg mature breast or back contour feathers from dark and light morph black sparrowhawks.

Feather type	PTCA peak area (mAU*s)
Light morph breast	No PTCA peak detected
Dark morph breast	511.7 ± 2.7
Light morph back	521.7 ± 4.0
Dark morph back	526.2 ± 10.4

To determine whether plumage polymorphism in this species is associated with DNA sequence variation in the coding region of the *MC1R* gene, an 851 bp portion of the predicted 945 bp coding region (including all nucleotide positions previously linked to color polymorphism in birds and mammals) was amplified from five dark and five light morph birds and sequenced. All 10 *MC1R* sequences obtained were identical, except for a single synonymous C/T transition in one of the light morph birds (Supplementary Fig. S2). Cloning and sequencing of full-length coding regions of *ASIP* from these same birds yielded identical sequences for all 10 individuals (Supplementary Fig. S3).

**Fig. 3.**
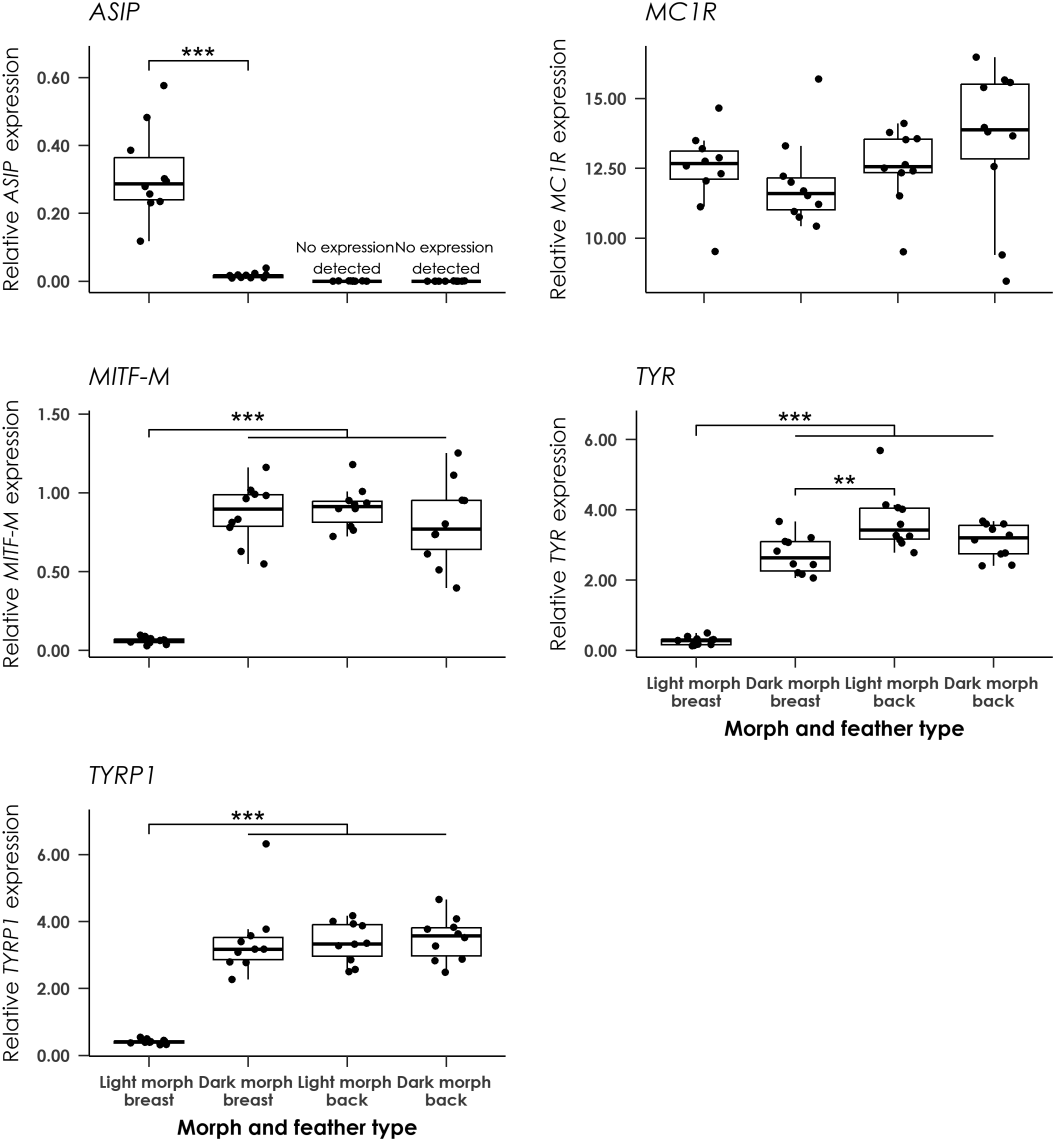
Box plots showing relative expression of the melanogenesis genes normalized to *GAPDH* expression in breast and back feathers from 10 light morph and 10 dark morph adult black sparrowhawks. Horizontal lines show mean values that are significantly different from each other as determined by post-hoc pairwise comparisons, with asterisks showing the level of significance (***P < 0.001; **P < 0.01). The boxes show the median and interquartile range, while the upper and lower whiskers extend to the highest or lowest value within 1.5 times the interquartile range.

RT-qPCR analysis was used to determine relative expression levels of the MC1R signaling pathway genes in developing breast and back feather tissue from 10 dark and 10 light morph birds. While no significant differences in *MC1R* expression (*F*_3,36_ = 1.21, *P* = 0.32, adjusted *R*^2^ = 0.016) were observed among feather types within or between morphs, *ASIP* expression was significantly higher (*F*_3,36_ = 54.39, *P* < 0.001, adjusted *R*^2^ = 0.80) in light morph versus dark morph breast feathers, and was not detectable in back feathers from either morph (Fig. 3). The downstream genes *MITF-M, TYR,* and *TYRP1* were expressed at significantly different levels among feather types within and between morphs (*MITF-M*: *F*_3,36_ = 52.08, *P* < 0.001, adjusted *R*^2^ = 0.80; *TYR*: *F*_3,36_ = 72.85, *P* < 0.001, adjusted *R*^2^ = 0.85; *TYRP1*: *F*_3,36_ = 45.77, *P* < 0.001, adjusted *R*^2^ = 0.78) (Fig. 3), and showed the opposite pattern of expression to *ASIP*, with significantly lower expression in light morph breast feathers than in dark morph breast feathers or in back feathers from either morph.

### The intensity of juvenile coloration is inversely correlated with *ASIP* expression

**Fig. 4.**
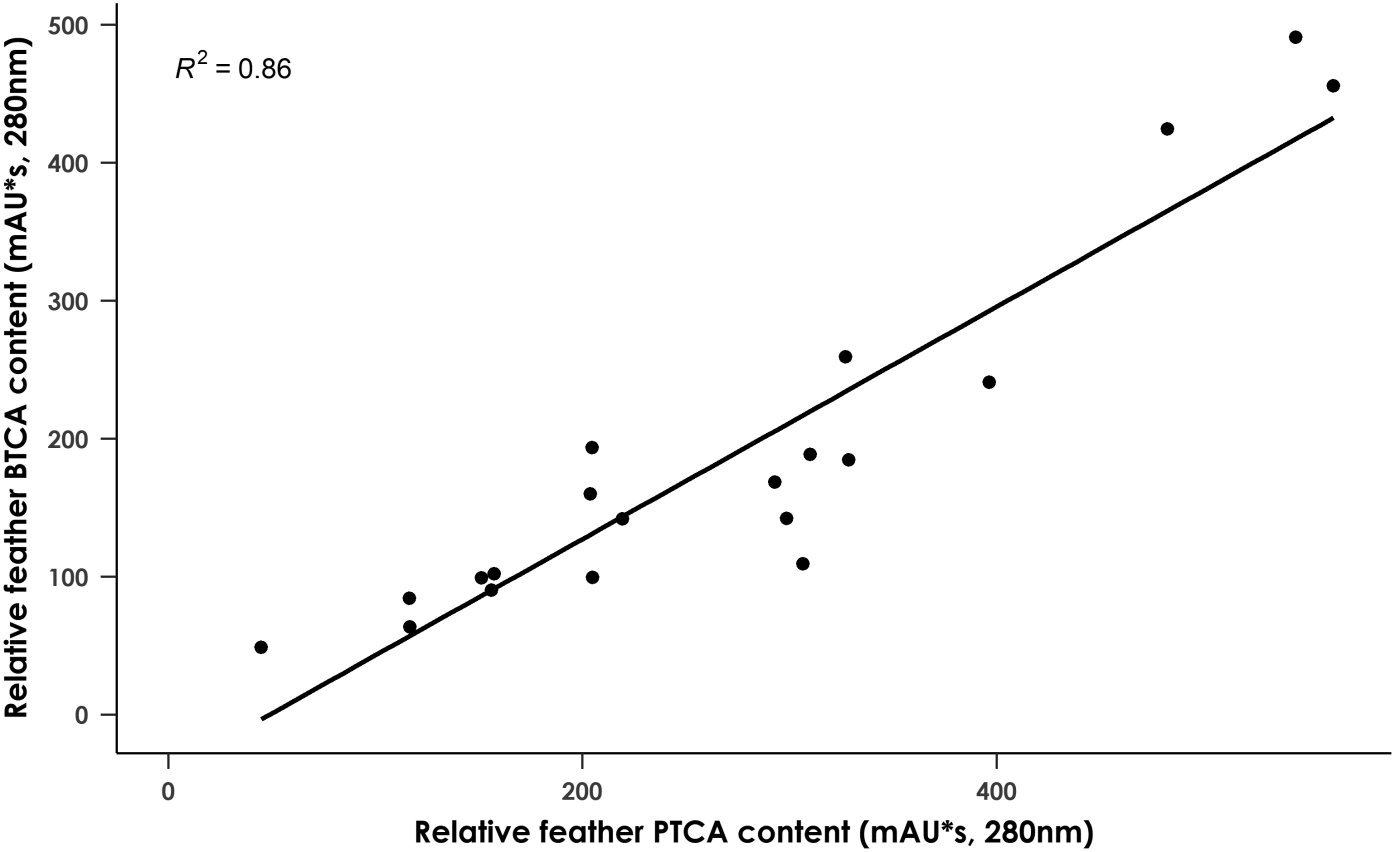
Scatter plot showing the correlation between PTCA and BTCA feather content in breast feathers from 20 juvenile black sparrowhawks. PTCA and BTCA content are significantly correlated (*χ*^2^_1, 20_ = 113.19, *P* < 0.001).

To determine the respective contributions of eumelanin and pheomelanin to juvenile breast coloration, PTCA and BTCA content were measured in breast feathers from 20 immature birds. In contrast to adult birds, no evidence was found for the existence of two discrete morphs in terms of melanin content. Instead, the variation in both PTCA and BTCA levels appeared to be continuous, and a highly significant positive correlation was observed between the two variables (*F*_1,18_ = 113.2, *P* < 0.001, adjusted *R*^2^ = 0.86) (Fig. 4). Thus, both eumelanin and pheomelanin contribute to plumage coloration in juvenile birds.

**Fig. 5.**
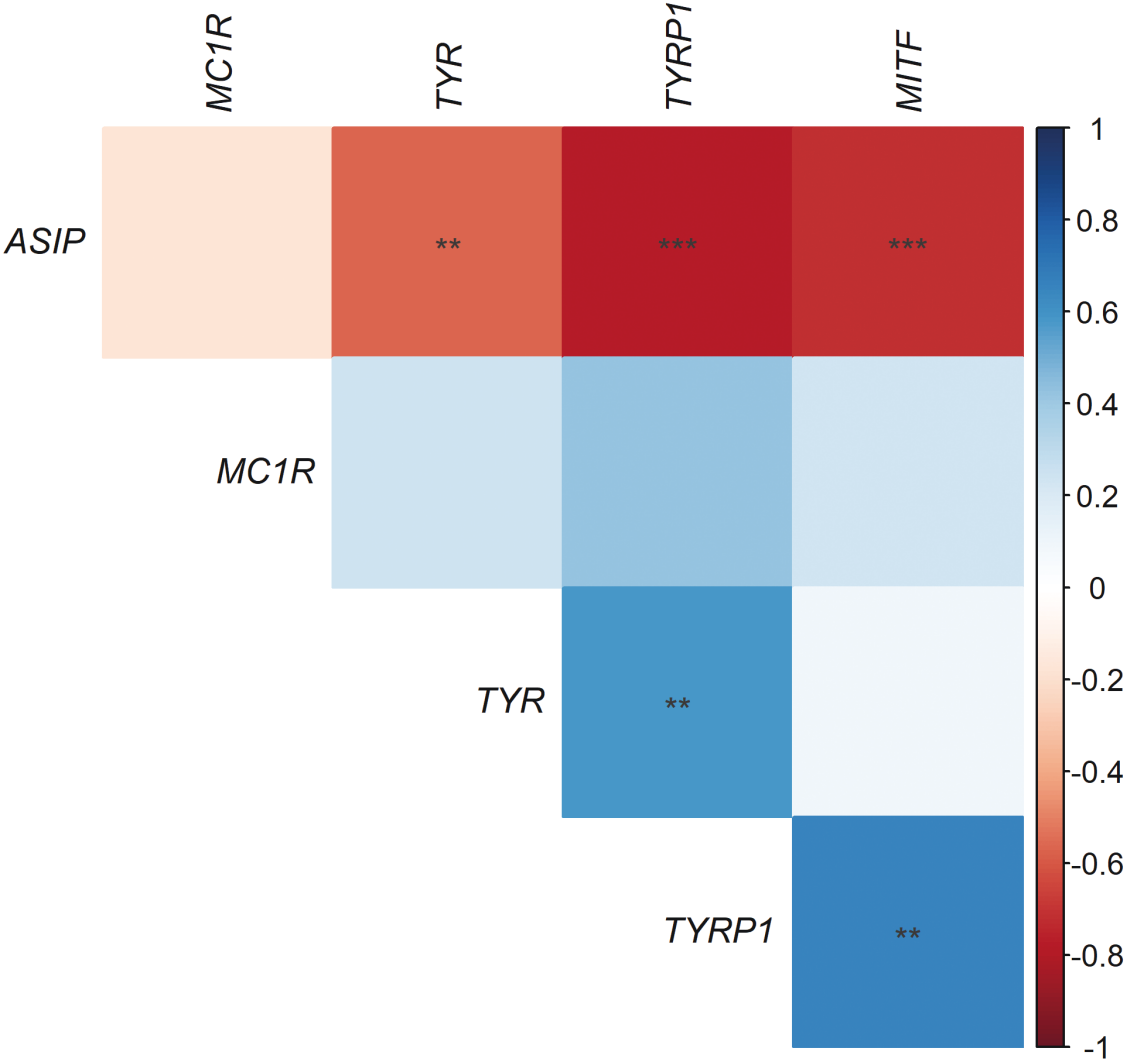
Heatmap showing the correlation between the relative level of expression of *ASIP, MC1R, MITF-M, TYR,* and *TYRP1* as quantified by RT-qPCR in developing breast feather tissue from 10 juvenile black sparrowhawks. The color indicates the strength of the correlation, and the scale shows Pearson’s correlation coefficient. Significant correlations are indicated by asterisks (**P < 0.01; ***P < 0.001).

**Fig. 6.**
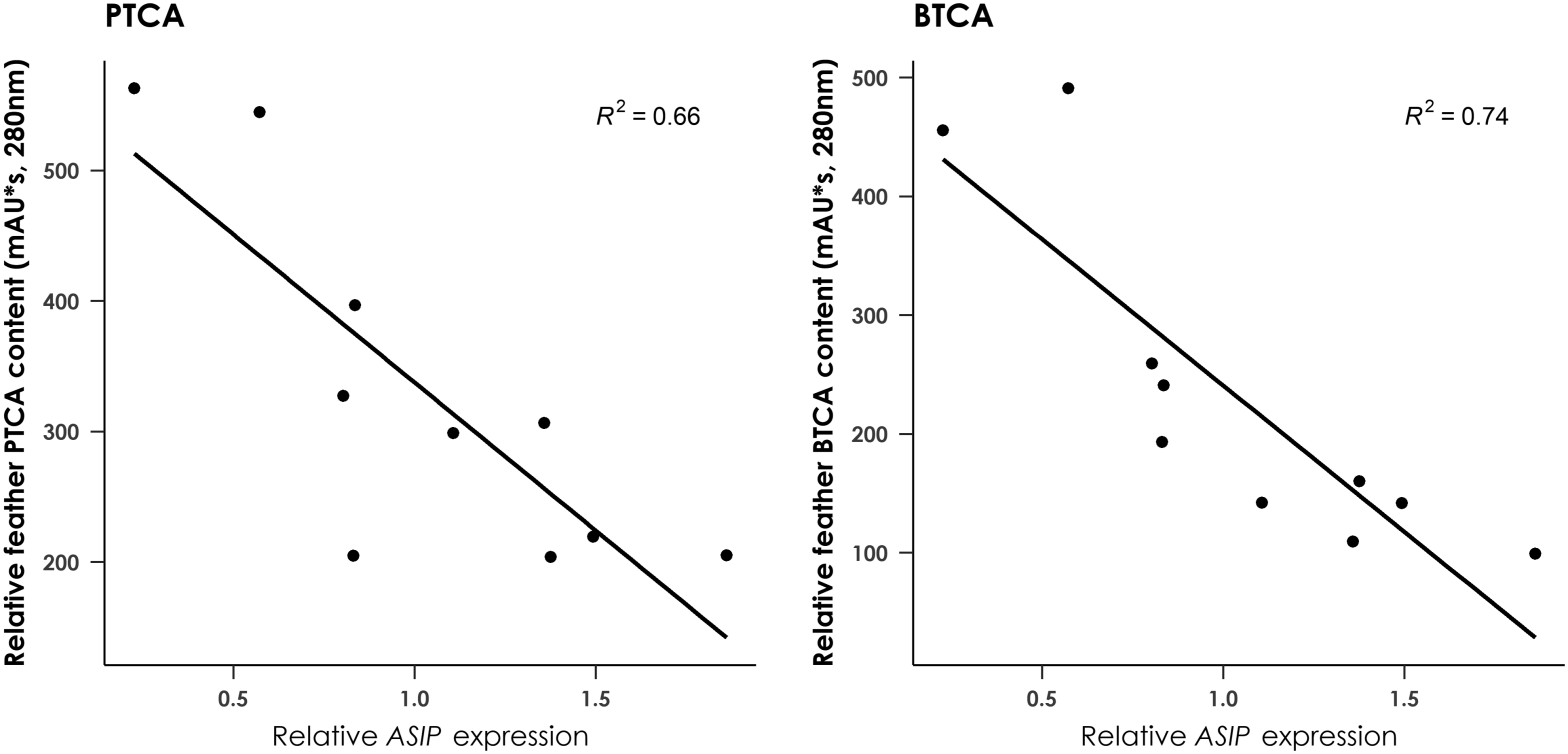
Scatter plots showing the relationship between PTCA (left) and BTCA (right) levels and *ASIP* expression in juveniles. *ASIP* expression is significantly negatively correlated with both PTCA (*F*_1,8_ = 15.49, *P* = 0.004) and BTCA (*F*_1,8_ = 23.03, *P* = 0.001) levels.

RT-qPCR analysis was used to compare the relative expression levels of the MC1R signaling pathway genes in developing breast and back feather tissue from 10 nestling birds with developing juvenile plumage. The uniformly darker back feathers displayed significantly lower *ASIP* expression (*F*_1,18_ = 26.62, *P* < 0.001, adjusted *R*^2^ = 0.57), with correspondingly higher expression of *MITF-M* (*F*_1,18_ = 11.03, *P* = 0.0038, adjusted *R*^2^ = 0.35), *TYR* (*F*_1,18_ = 7.02, *P* = 0.016, adjusted *R*^2^ = 0.24), and *TYRP1* (*F*_1,18_ = 24.08, *P* < 0.001, adjusted *R*^2^ = 0.55) than breast feathers, while no difference in *MC1R* expression was observed between feather types (*F*_1,18_ = 0.52, *P* = 0.48, adjusted *R*^2^ = −0.026) (Supplementary Fig. S4). To further explore the relationship between *ASIP* and expression levels of other MC1R signaling pathway genes and/or melanin content in developing breast feathers, we tested whether there was a correlation between these variables. Relative *ASIP* expression was not significantly correlated with *MC1R* expression (*R* = −0.18, *P* = 0.45) but was significantly negatively correlated with the expression levels of *MITF-M* (*R* = −0.72, *P* < 0.001), *TYR* (*R* = −0.57, *P* = 0.009), and *TYRP1* (*R* = −0.78, *P* < 0.001) (Fig. 5, Supplementary Table S3), while *TYRP-1* expression was significantly positively correlated with both *TYR* (*R* = 0.59, *P* = 0.007), and *MITF-M* (*R* = 0.66, *P* = 0.002) expression. Furthermore, *ASIP* expression was negatively correlated with both PTCA (*F*_1,8_ = 15.49, *P* = 0.004, adjusted *R*^2^ = 0.67) and BTCA (*F*_1,8_ = 23.03, *P* = 0.001, adjusted *R*^2^ = 0.71) levels (Fig. 6).

### Pheomelanin content of juvenile breast feathers is negatively correlated with the number of fault bars in flight feathers, but unrelated to plumage morph of parents or condition index

To determine whether juvenile plumage coloration is influenced by the morph (dark or light) of the parents, we tested for an association between breast feather PTCA and BTCA content and parental plumage morph. No significant relationship was detected for either PTCA (*F*_2,17_ = 1.10, *P* = 0.036, adjusted *R*^2^ = 0.01) or BTCA (*F*_2,17_ = 2.76, *P* = 0.092, adjusted *R*^2^ = 0.16), though this analysis is limited by the fact that only two of the adult pairs were LL (Supplementary Fig. S5). Similarly, there was no relationship between breast feather melanin content and the sex of the juvenile bird (PTCA: *F*_2,17_ = 0.08, *P* = 0.78, adjusted *R*^2^ = −0.05; BTCA: *F*_2,17_ = 0.13, *P* = 0.72, adjusted *R*^2^ = −0.05) (Supplementary Fig. S6).

**Fig. 7.**
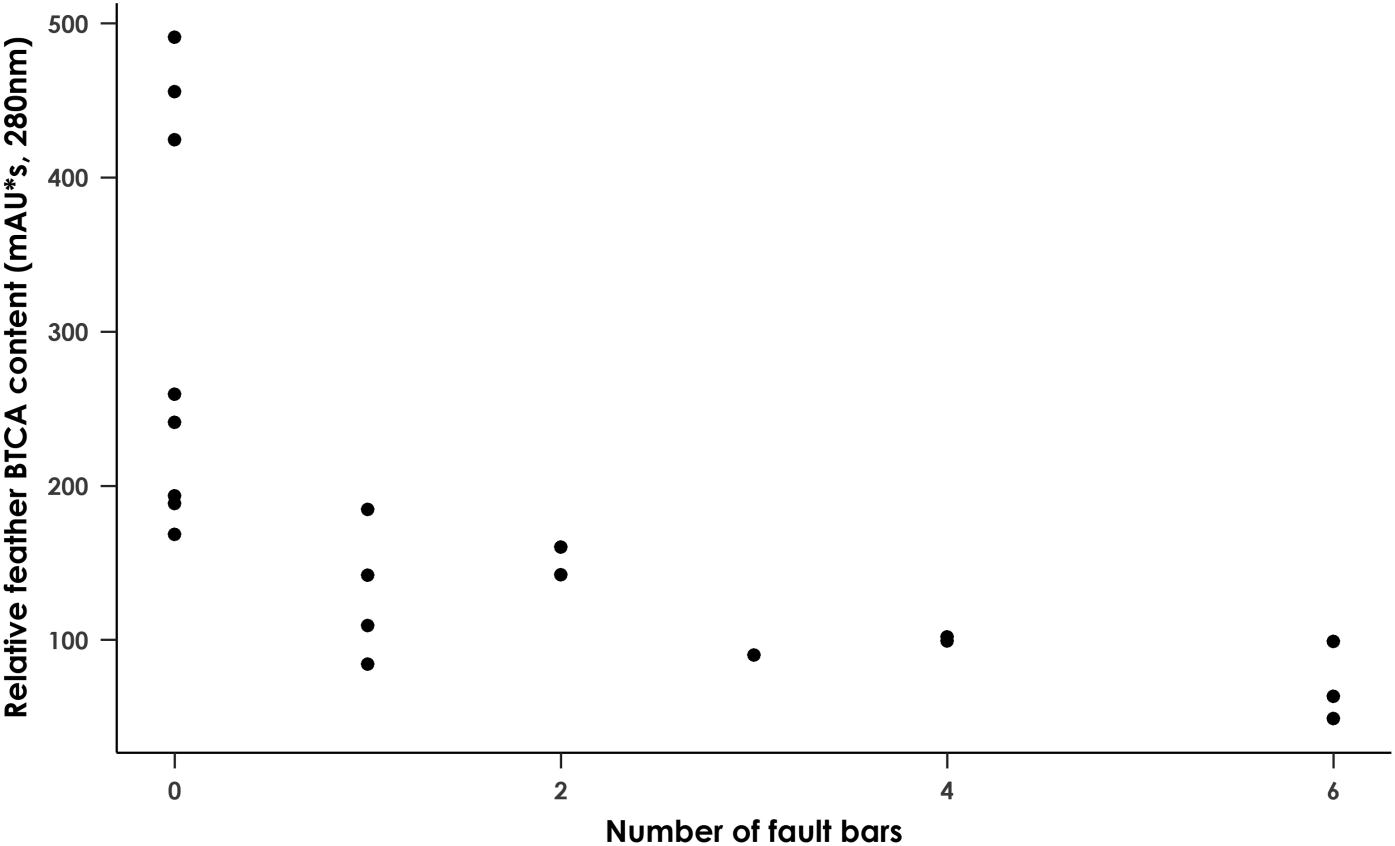
Scatter plot showing the relationship between the number of fault bars observed in flight feathers of 20 juvenile black sparrowhawks and BTCA content in developing breast feathers from the same individuals. Number of fault bars is significantly correlated with BTCA content (*χ*^2^_1, 20_ = 12.43, *P* < 0.001).

To investigate a possible role for environmental stress in juvenile pheomelanic pigmentation, we examined the relationship between BTCA content of developing breast feathers and the number of fault bars observed in the developing flight feathers, which could indicate stressful experiences during feather development, or condition index, which reflects nutritional status, in 20 nestling birds. While a significant negative correlation was observed between BTCA content and fault bar number (*F*_2,18_ = 6.65, *P* = 0.019, adjusted *R*^2^ = 0.23) (Fig. 7), there was no significant correlation between BTCA content and condition index (*F*_2,18_ < 0.001, *P* = 0.99, adjusted *R*^2^ = −0.06) (Supplementary Fig. S7).

## Discussion

As expected, given the pied plumage of adult black sparrowhawks, we found that the differences in coloration between the two morphs are driven solely by differences in eumelanin pigmentation. Light morph birds have no detectable eumelanin in their breast feathers, while dark morph breast feathers have eumelanin levels similar to those found in back feathers, albeit with a different pattern of distribution. While mutations in *MC1R* are often associated with melanistic plumage polymorphism in birds, this is not the case in the black sparrowhawk; identical *MCR1* sequences were obtained from four light and five dark morph birds, with a single synonymous transition present in the remaining light morph individual (Supplementary Fig. S2). Similarly, we found no evidence for any morph-specific polymorphisms in *ASIP* (Supplementary Fig. S3). The extent of variation in plumage between the two morphs is limited to the breast and underwing regions, and back feathers of both morphs contain the same amount of eumelanin (Table 1). This pattern is suggestive of ventral-specific differences in the regulation of melanin production, rather than a body-wide overproduction of this pigment due to constitutive activation of MC1R. Our qPCR analysis of the *MC1R* signaling pathway genes supports this hypothesis. While no differences in gene expression were observed between developing back feathers from the two morphs, we found a marked difference in gene expression in developing breast feathers. In light morph breast feathers, high expression of the MC1R antagonist *ASIP* was associated with low expression levels of the downstream genes involved in eumelanin production, *MITF-M*, *TYR*, and *TYR1P*. Conversely, low expression of *ASIP* in developing breast feathers from dark morph birds was associated with high expression of *MITF-M*, *TYR,* and *TYR1P*, and the same expression pattern was observed in the dark back feathers from both morphs (Fig. 3).

The light and dark plumage morphs of adult black sparrowhawks do not represent the only plumage variation in this species. Juvenile birds display the rufous coloration typical of the genus *Accipiter* (Ferguson-Lees et al. 2001), which varies in intensity between birds and is both eumelanin and pheomelanin-based, as is the case in the northern goshawk (Galván et al. 2019). While in this species pheomelanin levels are greatly reduced with the transition to adult plumage, in the black sparrowhawk pheomelanin appears to be completely absent in adults. As is the case in adult black sparrowhawks, increased *ASIP* expression was strongly correlated with decreased *MITF-M*, *TYR*, and *TYRP1* expression in the developing feathers of juvenile birds (Fig. 5). Eumelanin and pheomelanin levels were tightly correlated in juvenile feathers (Fig. 4), and so increased *ASIP* expression was strongly associated with a decrease in the levels of both pigments (Fig. 6).

The negative correlation observed between *ASIP* expression and eumelanin concentration in both adult and juvenile feathers is in line with the murine model of ASIP function, where ASIP acts as an agonist of the MC1R, blocking the activation of downstream melanogenesis genes including *TYR* and *MITF* (Lu et al. 1994). However, binding of ASIP to MC1R is classically associated with a switch to pheomelanin synthesis in mice, and studies in the Japanese quail have suggested that ASIP may perform a similar function in birds (Hiragaki et al. 2008; Nadeau et al. 2008). This is not the case in juvenile black sparrowhawks; levels of both eumelanin and pheomelanin decrease with increasing *ASIP* expression. Increased ASIP expression/activity is, however, not universally associated with pheomelanin production in birds. For example, in Silver Laced Wyandotte chickens, ASIP-coated beads inserted into the black region of a developing feather produce an area of white coloration, possibly through suppression of melanocyte differentiation (Lin et al. 2013), while transient over-expression of *ASIP* in Japanese quail embryos also resulted in hypopigmentation (Li et al. 2019).

Several studies have shown that the role of *ASIP* in the regulation of melanogenesis may be more complicated than previously thought, particularly in wild birds, which have been studied far less than domestic birds. The dark-eyed junco (*Junco hyemalis*) occurs in six geographically structured subspecific forms that vary markedly in plumage pigmentation, including the Oregon junco which produces both pheomelanin and eumelanin and the slate-colored junco where only eumelanin is present (Abolins-Abols et al. 2018). In both forms, the white ventral regions display elevated *ASIP* expression which is associated with low expression of *TYR* and *TYR1P* (Abolins-Abols et al. 2018). In the pheomelanin-producing Oregon junco, a 3-fold higher pheomelanin content in back versus flank feathers was correlated with significantly lower *ASIP* expression in developing back feathers (Abolins-Abols et al. 2018). Similarly, in the Eurasian nuthatch lower *ASIP* expression was associated with higher levels of pheomelanin production (Rodríguez-Martínez et al. 2019). Thus, in contrast to the murine system, increased *ASIP* expression can be associated with either a complete lack of pigmentation or with reduced pheomelanin production in birds, as seen in adult and juvenile black sparrowhawks, respectively. Which outcome occurs may depend on the capacity for pheomelanin production, which is apparently lacking in adult black sparrowhawks and slaty-colored juncos.

The variation in plumage intensity of the rufous juvenile black sparrowhawks appears to result from covariance in eumelanin and pheomelanin, whose levels are tightly linked and show a strong negative correlation with *ASIP* expression level. These results suggest that variation in juvenile plumage may be under tight genetic control, though we found no association with parental plumage morph. We found no correlation between body condition index, which reflects nutritional status and so could serve as a proxy for Cys availability, and pheomelanin content, which would be expected if pheomelanin was being produced to detoxify excess dietary Cys (Galván et al. 2012). However, a significant negative correlation between feather fault bar number and pheomelanin (and eumelanin) content was observed. Fault bars are thought to be associated with some form of chronic or, more likely, acute stress (Jovani and Rohwer 2017). In birds, stress response is largely mediated by glucocorticoid hormones, which increase quickly in stressful situations, causing behavioral modifications and energy mobilization (Sapolsky et al. 2000; Bonier et al. 2009, 2011). Elevated glucocorticoid levels may result in increased oxidative stress, with associated negative fitness costs (Costantini et al. 2011), and both chronic and acute stress have been shown to result in a greater level of oxidative stress (Lin et al. 2004; Costantini 2008; Marasco et al. 2017; Majer et al. 2019).

It is thus conceivable that the intensity of pheomelanic juvenile plumage in the black sparrowhawk is linked to the degree of oxidative stress experienced by the bird during feather development. Oxidative stress can cause depletion of GSH through efflux of excess glutathione disulphide (GSSG) from the cell to avoid toxicity, thus preventing it from being recycled to GSH by glutathione reductase (Lu 1999, 2009; Franco and Cidlowski 2012). This necessitates the de novo production of GSH to maintain a low GSSG:GSH ratio in order to prevent cell death (Nur et al. 2011), which in turn requires Cys. Pheomelanin production under conditions of oxidative stress may be undesirable, as it could leave individuals open to the detrimental effects of oxidative stress if it depletes Cys which could otherwise have been used for GSH production (Galván et al. 2012; Morgan et al. 2013). Thus, individuals suffering from a high level of oxidative stress, reflected in an increased number of fault bars, should have lower levels of pheomelanin, as we observed here. Interestingly, both *MC1R* and *ASIP* expression have been shown to be Cys responsive, increasing in Eurasian nuthatches fed a Cys-supplemented diet (Rodríguez-Martínez et al. 2019). Alternatively, variation in pheomelanic juvenile plumage in the black sparrowhawk could be linked to pleiotropic effects of the melanocortin system, with elevated levels of *ASIP* expression in lighter individuals linked to other behavioral and/or physiological functions mediated by the binding of ASIP to other melanocortin receptors (see Ducrest et al. 2008).

In both juvenile and light morph adult black sparrowhawks ventral contour feathers are lighter than dorsal contour feathers. This dorsoventral countershading is a common pattern throughout the animal kingdom, and has been linked to ventral-specific expression of *ASIP* resulting in lighter pigmentation versus the dorsal region in fish (Cerdá-Reverter et al. 2005), mammals (Vrieling et al. 1994), and domestic birds (Nadeau et al. 2008; Gluckman and Mundy 2017) where it is associated with downregulation of *TYR* and *TYR1P* expression. The observation that *ASIP* is alternatively spliced in mice, with the resulting transcripts containing different 5ʹ untranslated exons but identical protein-coding exons, led to the discovery that two regulatory regions can drive *ASIP* expression in mice, of which the distal promoter is only active in the ventral region (Vrieling et al. 1994). A similar regulatory mechanism operates in chickens where the transcription of the ventral-specific class 1A *ASIP* mRNA is driven only from the distal promoter (Oribe et al. 2012).

**Fig. 8.**
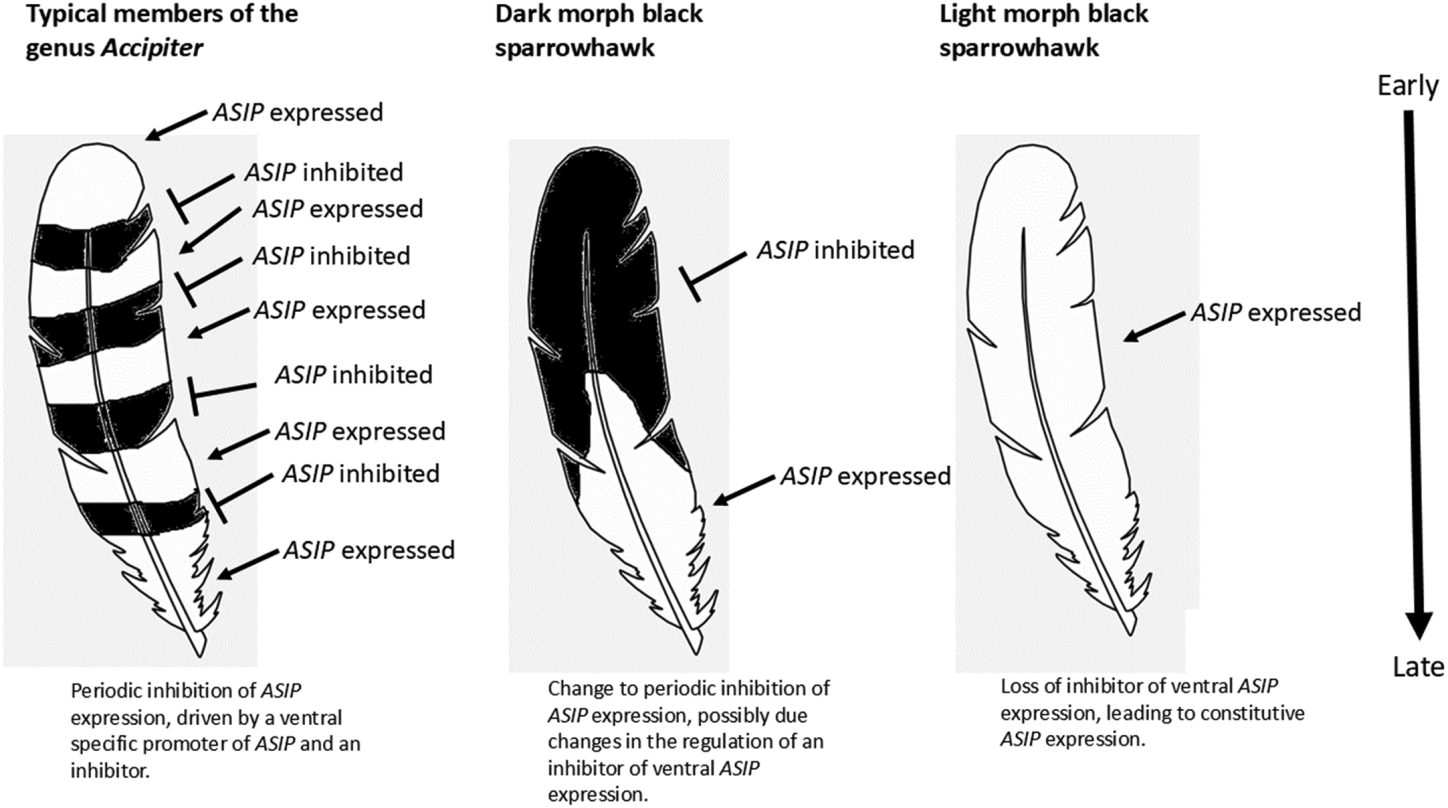
Hypothetical regulation of *ASIP* during the development of breast contour feathers in the black sparrowhawk, compared to possible patterns of *ASIP* expression in the development of barred contour feathers typical of the genus *Accipiter*. Note that the differences in expression are temporal, that is, *ASIP* is expressed during the development of differently pigmented feather regions as they develop from the feather bulb, not at different locations along the feather. The feather develops by growing from the base, with more distal regions having developed earlier than proximal ones.

Plumage morph in the black sparrowhawk is inherited in a simple Mendelian one locus, bi-allelic system, with the dark allele dominant over light (Nebel et al. 2021). While this inheritance pattern is common in species where the dark morph is associated with an overactive MC1R due to a polymorphism in the coding region of this gene (Mundy 2005), this is not the case in the black sparrowhawk. Instead, the evolution of plumage polymorphism in this species may tentatively be explained by invoking mutations that change the transcriptional activity from the ventral-specific *ASIP* promoter (Fig. 8). Unlike the black sparrowhawk, in most members of the genus *Accipiter* breast feathers are characterized by a strong barring pattern, most likely caused by pulses of melanin synthesis in the developing breast feathers (Louette 2000, 2006; Ferguson-Lees et al. 2001). Due to the role of ventral *ASIP* expression in countershading throughout the animal kingdom, the barring pattern seen in the breast feathers of members of the genus *Accipiter* might result from repeated cycles of activation and repression of *ASIP* expression in the ventral region. This would be somewhat similar to the agouti phenotype in mice (a pale band on an otherwise black hair) which results from a short burst of *ASIP* expression during the mid-point of the hair growth cycle (Millar et al. 1995). However, in the black sparrowhawk, a change in the temporal expression of a transcriptional repressor of the ventral promoter may have occurred, such that *ASIP* is repressed for an extended period of time at the beginning of feather development, and activated only towards the end of this period, thus resulting in the black-tipped breast feathers with white base observed in the dark morph birds. The recessive nature of the light morph phenotype is consistent with a loss-of-function mutation and could have evolved subsequently through homozygosity for a loss-of-function mutation in this transcriptional repressor. The resulting constitutive expression of *ASIP* would then inhibit melanin production by downregulation of *MITF*, *TYR,* and *TRY1P*. Finally, it is conceivable that the evolutionary advantages associated with dorsoventral countershading might also explain the current prominence of the homozygous recessive light morph phenotype in the population, as these birds have an advantage when foraging under high-light conditions (Tate et al. 2016).

In conclusion, plumage morph in adult black sparrowhawks is determined by the pattern of eumelanin deposition, underpinned by differences in the expression of melanogenesis genes associated with the MC1R pathway. High levels of ventral-specific expression of *ASIP* in light morph birds are associated with significantly lower expression of the downstream melanogenesis genes *MITF*, *TYR*, and *TYR1P* and the absence of eumelanin. Feathers of adult birds contain only eumelanin, whereas those of juvenile birds contain both eumelanin and pheomelanin. The regulatory mechanism behind this, and the reason for the tight coupling of eumelanin and pheomelanin levels in juvenile birds is currently unknown. Nevertheless, the regulation of both dorsoventral countershading and variation in plumage intensity of breast feathers is likely to be similar to that in adults, with higher expression of *ASIP* in breast feathers and lighter feathers downregulating downstream MC1R melanogenesis genes and thus melanin production.

## Supplementary material

Supplementary material is available at *Journal of Heredity* online.

esae068_suppl_Supplementary_Materials

## Data Availability

The DNA sequences generated in this study are deposited in Genbank: MC1R (ON209113), ASIP (ON209114), MITF (ON209115), TYR (ON209116), TYRP1 (ON209117), and GAPDH (ON209118). The HPLC and qPCR data generated during this project, the R code used to analyze it and the sample metadata are available at https://doi.org/10.25375/uct.25627092.
