# Supplementary material for: Plumage polymorphism in the black sparrowhawk (Accipiter melanoleucus) is strongly associated with the expression level of agouti signaling protein
Source: J Hered. 2024 Nov 18;116(3):193–207. doi: 10.1093/jhered/esae068 (PMC12130442; doi:10.1093/jhered/esae068)
Supplement: esae068_suppl_Supplementary_Materials [file esae068_suppl_supplementary_materials.pdf]

SUPPORTING INFORMATION

Table S1. Primers used to amplify full- or partial-length coding sequences of melanogenesis and reference genes from black sparrowhawk genomic DNA or cDNA (derived from RNA extracted from developing contour feathers). Associated annealing temperatures (Ta) used in PCR reactions and product size, including whether partial (P) or full-length (F) coding sequence are provided.

Gene	Primers	Primer sequence	Ta	Product size (bp)
MC1R	MC1Rf	5′ ATGCCAGTGAGGGCAACCA 3′	68°C	851 (P)
	MC1Rr	5′ CTGGCTCCGGAAGGCATAGAT 3′		
ASIP	ASIPf	5′ ATGACAATGGAATTTTTCCTCCCCA 3′	65°C	415 (F)
	ASIPr	5′ TGGGTTTAACATTAGACATTGGCAGA 3′		
MITF	MITFf	5′ ATGCTGGAAATGCTTGAGT 3′	62°C	1142 (F)
	MITFr	5′ GTGTCCTCCATGCTCACG 3′		
TYR	TYRf	5′ TAGTCATCCTTCAGCCATCCAC 3′	65°C	997 (F)
	TYRr	5′ TTTCGGAAACTGTAATTGGCCA 3′		
TYRP1	TYRP1f	5′ GGCGAGTGCTGTGTGAAAATAT 3′	65°C	88 (P)
	TYRP1r	5′ GGATTTCTTCGGATGGGACC 3′		
GAPDH	GAPDHf	5′ CTGTCAAGGCTGAGAATGG 3′	65°C	280 (P)
	GAPDHr	5′ CAAGAGGCATTGCTGACA 3′		

Table S2. Primers used in RT-qPCR and their expected product sizes using black sparrowhawk gDNA or cDNA as template.

**Gene**	**Primers**	**Primer sequence**	**Product size (cDNA)**	**Product size (gDNA)**
MC1R	MC1R qF	5′ GATGACGTTGTCCATGTGG 3′	150 bp	150 bp
	MC1R qR	5′ ACCCACGTACTACTTCATCT 3′		
ASIP	ASIP qF	5′ TCCCAGAAAGTCAGCAGG 3′	120 bp	c. 1700 bp
	ASIP qR	5′ TTGAAGTTTGGCACACAGTC 3′		
MITF	MITF qF	5′ CATTATCAGGTGCAGACTCAC 3′	90 bp	None (intron spanning primers)
	MITF qR	5′ GTGGTAGAGAGGTACTGCTT 3′		
TYR	TYR qF	5′ CTCATGGCAGGTAATTTGTAC 3′	105 bp	None (intron spanning primers)
	TYR qR	5′ ATTGTTCCCAGGATTTCGTAG 3′		
TYRP1	TYRP1 qF	5′ GGCGAGTGCTGTGTGAAAATAT 3′	87 bp	c. 1500 bp
	TYRP1 qR	5′ GGATTTCTTCGGATGGGACC 3′		
GAPDH	GAPDH qF	5′ CTGTCAAGGCTGAGAATGG 3′	280 bp	None (intron spanning primers)
	GAPDH qR	5′ CAAGAGGCATTGCTGACA 3′		

Table S3. Correlation table and associated p values (Pearson) showing the relationships between expression levels of the melanogenesis genes in juvenile breast feathers.

ASIP	***TYR***	***MITF***	***TYRP-1***	***MC1R***	
	R = -0.57<br>p = 0.009	R = -0.72<br>p < 0.001	R = -0.78<br>p < 0.001	R = -0.18<br>p = 0.45	***ASIP***
		R = 0.09<br>p = .070	R = 0.59<br>p = 0.007	R = 0.25<br>p = 0.30	***TYR***
			R = 0.66<br>p = 0.007	R = 0.23<br>p = 0.32	***MITF***
				R = 0.42<br>p = 0.07	***TYRP-1***
					MC1R

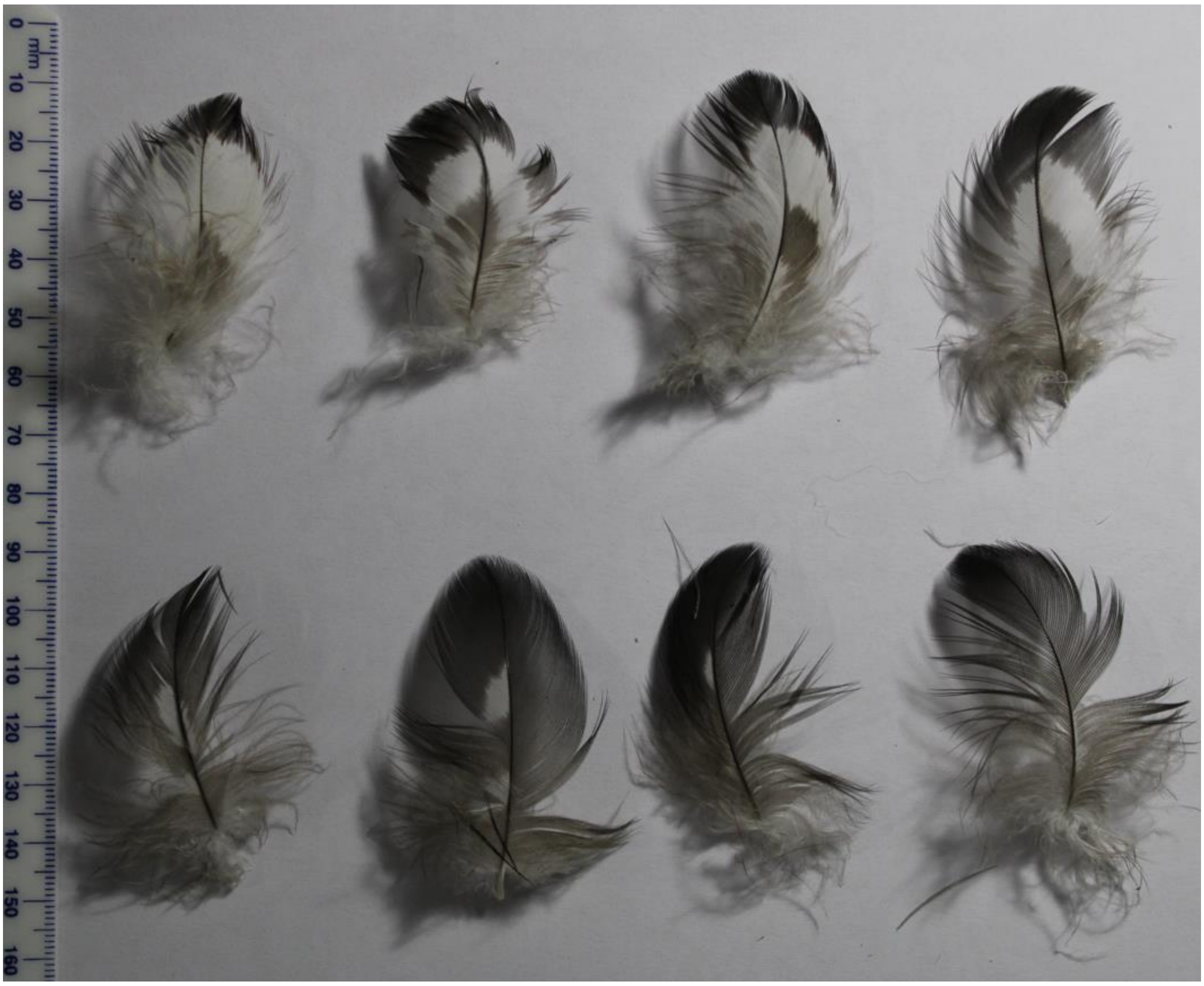

Figure S1. Variation in adult dark morph black sparrowhawk breast feathers. In adult dark morphs, breast feathers can vary in colouration from almost completely white with a small black tip (top left) to completely black (bottom right). Note that back feathers are uniformly black, as in the light morph. Ruler for scale (mm).

```
                        10        20        30        40        50        60        70        80        90       100
                ....|....|....|....|....|....|....|....|....|....|....|....|....|....|....|....|....|....|....|....|
Dark 1 MC1R     GAGCAATGCCGGGGCCGGCAGCGCCTGGTGCCAGGGGCTCAACATTCCCAGCGAACTATTCCTCACGCTGGGACTGGTGAGCCTGGTGGAGAACCTGCTG
Dark 2 MC1R     ....................................................................................................
Dark 3 MC1R     ....................................................................................................
Dark 4 MC1R     ....................................................................................................
Dark 5 MC1R     ....................................................................................................
Light 1 MC1R    ....................................................................................................
Light 2 MC1R    ....................................................................................................
Light 3 MC1R    .........................................................................T..........................
Light 4 MC1R    ....................................................................................................
Light 5 MC1R    ....................................................................................................

                       110       120       130       140       150       160       170       180       190       200
                ....|....|....|....|....|....|....|....|....|....|....|....|....|....|....|....|....|....|....|....|
Dark 1 MC1R     GTGGTGGCTGCCATCCTGAAGAACAGGAACCTGCACTCACCCACGTACTACTTCATCTGCTGCCTGGCCGTCTCCGACATGCTGGTGAGCGTCAGCAACC
Dark 2 MC1R     ....................................................................................................
Dark 3 MC1R     ....................................................................................................
Dark 4 MC1R     ....................................................................................................
Dark 5 MC1R     ....................................................................................................
Light 1 MC1R    ....................................................................................................
Light 2 MC1R    ....................................................................................................
Light 3 MC1R    ....................................................................................................
Light 4 MC1R    ....................................................................................................
Light 5 MC1R    ....................................................................................................

                       210       220       230       240       250       260       270       280       290       300
                ....|....|....|....|....|....|....|....|....|....|....|....|....|....|....|....|....|....|....|....|
Dark 1 MC1R     TGGCAGAGACACTCTTCATGCTGCTGATGGAGCACGGCGTGCTGGTGGTCCACACCAGCATCGTCCGCCACATGGACAACGTCATCGACATGCTCATCTG
Dark 2 MC1R     ....................................................................................................
Dark 3 MC1R     ....................................................................................................
Dark 4 MC1R     ....................................................................................................
Dark 5 MC1R     ....................................................................................................
Light 1 MC1R    ....................................................................................................
Light 2 MC1R    ....................................................................................................
Light 3 MC1R    ....................................................................................................
Light 4 MC1R    ....................................................................................................
Light 5 MC1R    ....................................................................................................
```

```
                      310       320       330       340       350       360       370       380       390       400
                  ....|....|....|....|....|....|....|....|....|....|....|....|....|....|....|....|....|....|....|....|
Dark 1 MC1R       CAGCTCTGTCGTGTCCTCCCTCTCCTTCCTGGGGGTCATCGCTGTGGACCGCTACATCACCATCTTCTACGCCCTGCGCTACCACAGCATCATGACGCTG
Dark 2 MC1R       ....................................................................................................
Dark 3 MC1R       ....................................................................................................
Dark 4 MC1R       ....................................................................................................
Dark 5 MC1R       ....................................................................................................
Light 1 MC1R      ....................................................................................................
Light 2 MC1R      ....................................................................................................
Light 3 MC1R      ....................................................................................................
Light 4 MC1R      ....................................................................................................
Light 5 MC1R      ....................................................................................................

                      410       420       430       440       450       460       470       480       490       500
                  ....|....|....|....|....|....|....|....|....|....|....|....|....|....|....|....|....|....|....|....|
Dark 1 MC1R       CAACGGGCCGTGGTGACCATGGCCAGCATCTGGCTGACCAGCACTGTCTCCAGCACTGTCTTCATCACCTACTGCCACAACAATGCCATCCTCCTCTGCC
Dark 2 MC1R       ....................................................................................................
Dark 3 MC1R       ....................................................................................................
Dark 4 MC1R       ....................................................................................................
Dark 5 MC1R       ....................................................................................................
Light 1 MC1R      ....................................................................................................
Light 2 MC1R      ....................................................................................................
Light 3 MC1R      ....................................................................................................
Light 4 MC1R      ....................................................................................................
Light 5 MC1R      ....................................................................................................

                      510       520       530       540       550       560       570       580       590       600
                  ....|....|....|....|....|....|....|....|....|....|....|....|....|....|....|....|....|....|....|....|
Dark 1 MC1R       TCATCGGCTTCTTCCTCTTCATGCTGGTCCTCATGCTGGTGCTCTACATCCACATGTTCACCCTGGCCCGCCACCACCTCCGCAGCATCTCCAGGCAGCA
Dark 2 MC1R       ....................................................................................................
Dark 3 MC1R       ....................................................................................................
Dark 4 MC1R       ....................................................................................................
Dark 5 MC1R       ....................................................................................................
Light 1 MC1R      ....................................................................................................
Light 2 MC1R      ....................................................................................................
Light 3 MC1R      ....................................................................................................
Light 4 MC1R      ....................................................................................................
Light 5 MC1R      ....................................................................................................
```

```
                  610       620       630       640       650       660       670       680       690       700
              ....|....|....|....|....|....|....|....|....|....|....|....|....|....|....|....|....|....|....|....|
Dark 1 MC1R   GAAGCAGCCCACCGTCTACCATGACAGCAGCCTGAAGGGAGCTGTCACGCTCACCATCCTCCTGGGCGTCTTCTTCATCTGCTGGGGGCCCTTCTTCTTC
Dark 2 MC1R   ....................................................................................................
Dark 3 MC1R   ....................................................................................................
Dark 4 MC1R   ....................................................................................................
Dark 5 MC1R   ....................................................................................................
Light 1 MC1R  ....................................................................................................
Light 2 MC1R  ....................................................................................................
Light 3 MC1R  ....................................................................................................
Light 4 MC1R  ....................................................................................................
Light 5 MC1R  ....................................................................................................

                  710       720       730       740       750       760       770       780       790       800
              ....|....|....|....|....|....|....|....|....|....|....|....|....|....|....|....|....|....|....|....|
Dark 1 MC1R   CACCTCATCCTCATTGTCACCTGTCCCACCAACCCCTTCTGCACCTGCTTCTTCAGCTACTTCAACCTCTTCCTCATCCTCATCATCTGTAACTCAGTCA
Dark 2 MC1R   ....................................................................................................
Dark 3 MC1R   ....................................................................................................
Dark 4 MC1R   ....................................................................................................
Dark 5 MC1R   ....................................................................................................
Light 1 MC1R  ....................................................................................................
Light 2 MC1R  ....................................................................................................
Light 3 MC1R  ....................................................................................................
Light 4 MC1R  ....................................................................................................
Light 5 MC1R  ....................................................................................................

                  810
              ....|....|.
Dark 1 MC1R   TCGACCCCCTC
Dark 2 MC1R   ...........
Dark 3 MC1R   ...........
Dark 4 MC1R   ...........
Dark 5 MC1R   ...........
Light 1 MC1R  ...........
Light 2 MC1R  ...........
Light 3 MC1R  ...........
Light 4 MC1R  ...........
Light 5 MC1R  ...........
```

Figure S2. Alignment of *MC1R* amplicon nucleotide sequences from five dark-morph adult and five light-morph adult black sparrowhawks. Note that the primers have been trimmed from these sequences (final length = 811 bp).

```
                        10        20        30        40        50        60        70        80        90        100
                ....|....|....|....|....|....|....|....|....|....|....|....|....|....|....|....|....|....|....|....|
Dark 1 ASIP    GGATGGAAAGTAAGAACCTCTTCCTAAGCCTATTGCTCTGCTACAGTTTGCTCAGAGCTGCAGATTCTTATATGGTAATTGAAGAGAAGACAGAATGCAA
Dark 2 ASIP    ....................................................................................................
Dark 3 ASIP    ....................................................................................................
Dark 4 ASIP    ....................................................................................................
Dark 5 ASIP    ....................................................................................................
Light 1 ASIP   ....................................................................................................
Light 2 ASIP   ....................................................................................................
Light 3 ASIP   ....................................................................................................
Light 4 ASIP   ....................................................................................................
Light 5 ASIP   ....................................................................................................

                       110       120       130       140       150       160       170       180       190       200
                ....|....|....|....|....|....|....|....|....|....|....|....|....|....|....|....|....|....|....|....|
Dark 1 ASIP    CCTATCAAGCAGCAGCAACAACATGAATCTCTCGGATCTCCCACCCATCTCCATAGTAGATCTAACTAGAAGATCCCAGAAAGTCAGCAGGAAAGAGGCA
Dark 2 ASIP    ....................................................................................................
Dark 3 ASIP    ....................................................................................................
Dark 4 ASIP    ....................................................................................................
Dark 5 ASIP    ....................................................................................................
Light 1 ASIP   ....................................................................................................
Light 2 ASIP   ....................................................................................................
Light 3 ASIP   ....................................................................................................
Light 4 ASIP   ....................................................................................................
Light 5 ASIP   ....................................................................................................

                       210       220       230       240       250       260       270       280       290       300
                ....|....|....|....|....|....|....|....|....|....|....|....|....|....|....|....|....|....|....|....|
Dark 1 ASIP    GAGAACAGGAAATCTTCCAAGAAAAAAGCTCAACCAAAGACACCTCCAAAACCAAGGCCTACACCAGCTGCTGACTGTGTGCCAAACTTCAAAACCTGCA
Dark 2 ASIP    ....................................................................................................
Dark 3 ASIP    ....................................................................................................
Dark 4 ASIP    ....................................................................................................
Dark 5 ASIP    ....................................................................................................
Light 1 ASIP   ....................................................................................................
Light 2 ASIP   ....................................................................................................
Light 3 ASIP   ....................................................................................................
Light 4 ASIP   ....................................................................................................
Light 5 ASIP   ....................................................................................................
```

```
                     310       320       330       340       350       360
                ....|....|....|....|....|....|....|....|....|....|....|....|...
Dark 1 ASIP     AACCACAGTTGAATTCATGTTGTAACTACTGTGCTTTGTGCAAATGCCGAATTTTTCAGACAA
Dark 2 ASIP     ...............................................................
Dark 3 ASIP     ...............................................................
Dark 4 ASIP     ...............................................................
Dark 5 ASIP     ...............................................................
Light 1 ASIP    ...............................................................
Light 2 ASIP    ...............................................................
Light 3 ASIP    ...............................................................
Light 4 ASIP    ...............................................................
Light 5 ASIP    ...............................................................
```

Figure S3. Alignment of *ASIP* amplicon nucleotide sequences from five dark-morph adult and five light-morph adult black sparrowhawks. Note that the primers have been trimmed from these sequences (final length = 363 bp).

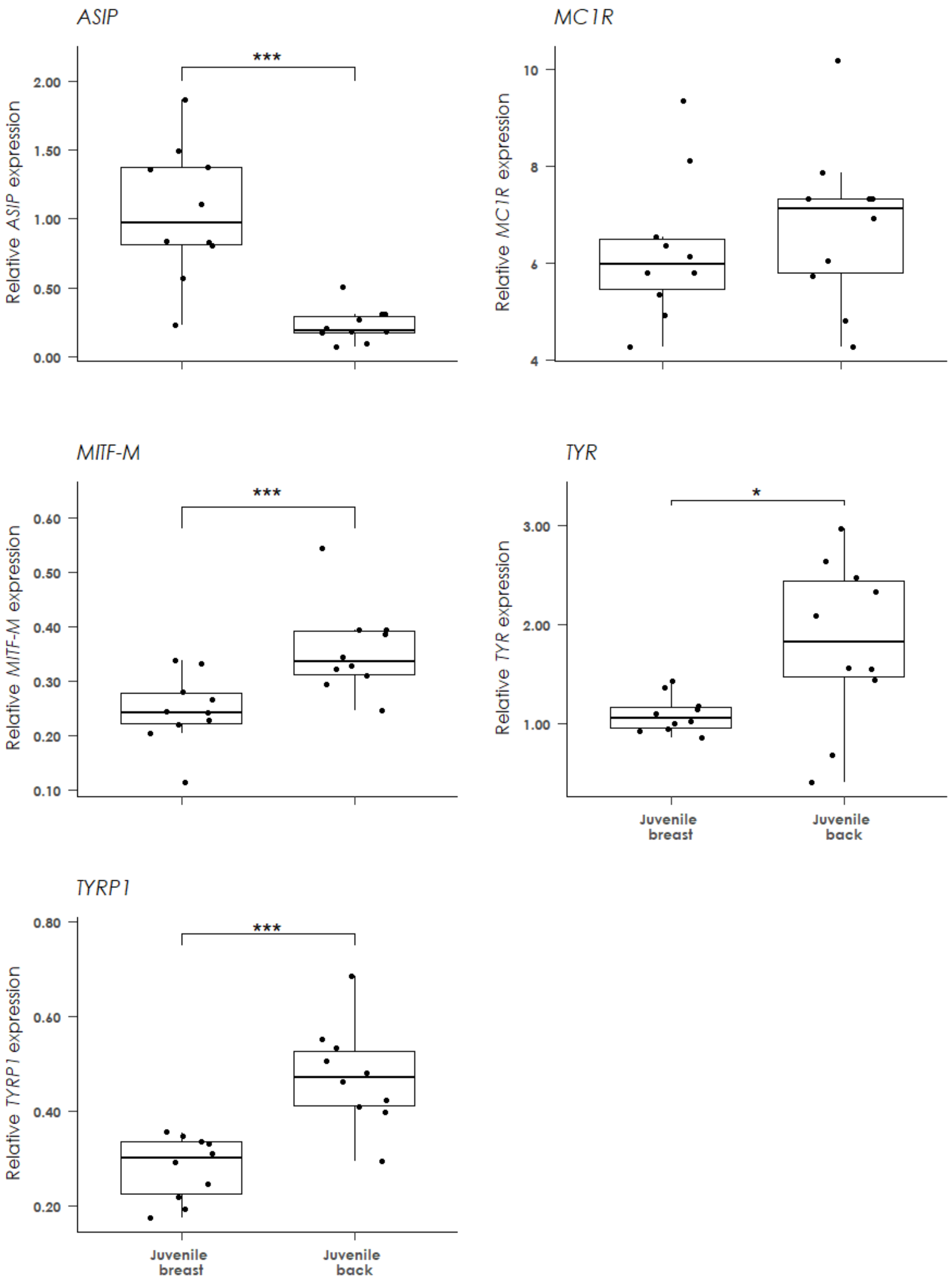

Figure S4. Box plots showing relative expression of melanogenesis genes normalised to GAPDH expression in breast and back feathers from ten juvenile black sparrowhawks. Horizontal lines show mean values that are significantly different to each other, with asterisks showing level of significance (*** : $p < 0.001$; ** : $p < 0.01$; * : $p<0.05$). The boxes show median and interquartile range, while the upper and lower whiskers extend to the highest or lowest value within 1.5 times the interquartile range.

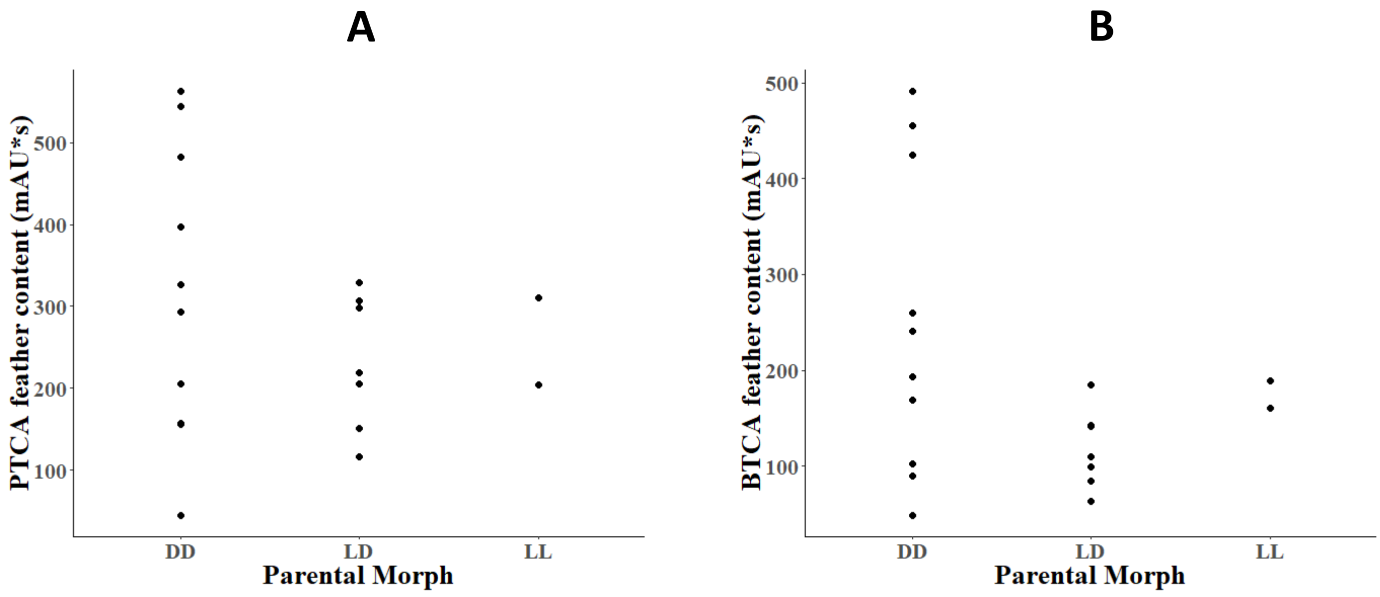

Figure S5. The relationship between parental morph and PTCA (A) and BTCA (B) levels in developing breast feathers from 20 juvenile black sparrowhawks. Parental morph DD signifies a pair in which both parents were dark morph, LD a pair in which one parent was light morph and one was dark, and LL a pair in which both parents were light morph.

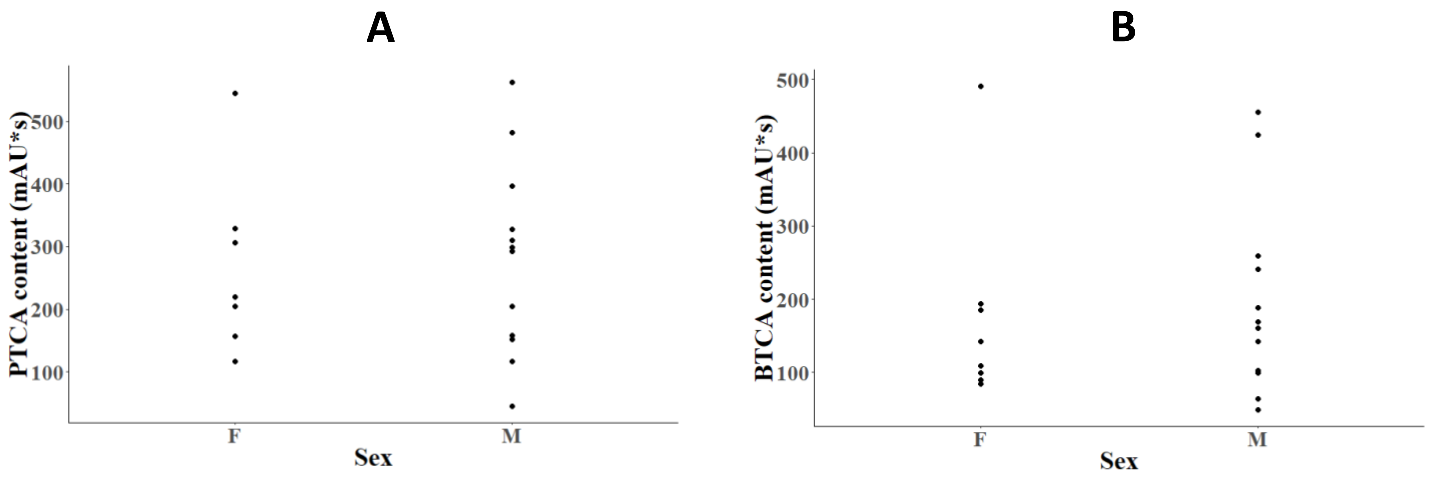

Figure S6. The relationship between sex (F = female and M = male) and PTCA (A) and BTCA (B) levels in developing breast feathers from 20 juvenile black sparrowhawks (8 females and 12 males).

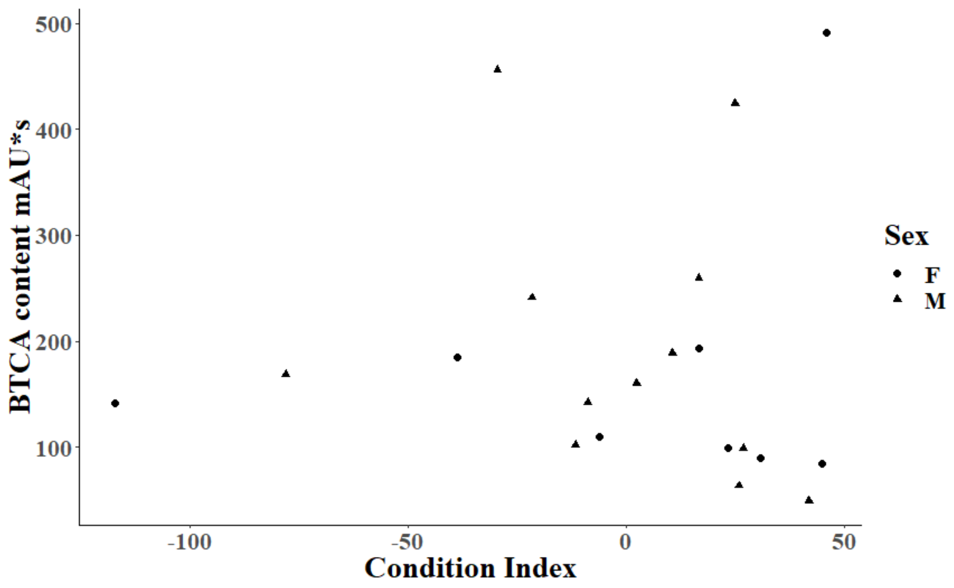

Figure S7. The relationship between condition index and breast feather BTCA content in 20 juvenile black sparrowhawks. Sex of the individuals, Female (F, 8 individuals) or Male (M, 12 individuals), is also shown.
